# Attrition and delays before treatment initiation among patients with MDR-TB in China (2006-13): Magnitude and risk factors

**DOI:** 10.1371/journal.pone.0214943

**Published:** 2019-04-08

**Authors:** Caihong Xu, Renzhong Li, Hemant Deepak Shewade, Kathiresan Jeyashree, Yunzhou Ruan, Canyou Zhang, Lixia Wang, Hui Zhang

**Affiliations:** 1 National Center for Tuberculosis Control and Prevention, China Center for Disease Control (CDC), Beijing, China; 2 International Union Against Tuberculosis and Lung Disease (The Union), South-East Asia Office, New Delhi, India; 3 International Union Against Tuberculosis and Lung Disease (The Union), Paris, France; 4 Karuna Trust, Bengaluru, India; 5 Velammal Medical College Hospital and Research Institute, Madurai, India; University of Liverpool, UNITED KINGDOM

## Abstract

**Background:**

China’s national tuberculosis programme does not have cohort wise information regarding attrition and delays in the multidrug resistant tuberculosis (MDR-TB) diagnosis and treatment pathway.

**Objective:**

Under the Global Fund programmatic management of drug-resistant TB (2006–13), we assessed the attrition and delay in the pathway and the factors associated.

**Methods:**

Cohort study involving secondary programme data. All patients identified as presumptive MDR-TB (defined as i) previously treated TB patients which included recurrent TB, return after loss to follow up, treatment after failure and ii) new TB patients that were non-converters at three months of treatment or in close contact with a known MDR-TB patient) during October 2006 to June 2013 were eligible for phenotypic drug susceptibility testing (DST). Pre-diagnosis attrition (presumptive MDR-TB not undergoing culture and DST) and pre-treatment attrition (confirmed MDR-TB patients not initiated on treatment) was calculated. Diagnosis delay was the time interval from DST eligibility to DST result, treatment initiation delay was fom DST result to treatment initiation and total delay was from DST eligbility to treatment initiation. Factors associated with attrition and delay were identified using log binomial regression and linear regression, respectively.

**Results:**

Of 78 564 presumptive MDR-TB patients, 2 470 (3.1%) underwent pre-diagnosis attrition. Of 9 283 MDR-TB patients, 3 361 (36.2%) underwent pre-treatment attrition. Median(IQR) diagnosis delay was 84 (64, 114) days; treatment initation delay was 23(6,68) days and total delay was 117(77,187) days. Long diagnosis delay was an independent predictor of pre-treatment attrition in a dose response relationship. While pre-treatment attrition was less likely among presumptive criterion ‘previously treated’ and with increasing time period, it was more likey among elderly and in east and west region. While the diagnosis delay increased with time period, treatment initiation delay and total delay reduced with time period. Short diagnosis delay was associated with west region, smear negative patients and presumptive criterion ‘treatment after lost to follow up’. Short treatment initiation delay was associatied with east and west regions while long treatment initiation delay was associated with elderly and presumptive criterion ‘recurrent TB’. Total delay predictors were similar to treatment initiation delay. In addition, short total delay was associated with presumptive criterion ‘treatment after failure’.

**Conclusion:**

The diagnosis and treatment delay were long and the pre-treatment attrition was considerable high. Long diagnosis delay is likely to predict pre-treatment attrition.

## Introduction

Multidrug-resistant TB (MDR-TB), defined as resistance to at least isoniazid and rifampicin, along with rifampicin-resistant tuberculosis (RR-TB), is a major public health concern in many countries and threatens global attempts to end TB [1]. Timely identification and prompt treatment initiation of MDR-TB patients are crucial to prevent the transmission of infection and reduce related morbidity and mortality.

Majority of MDR-TB patients are lost in the diagnosis and treatment pathway. Globally in 2017, there were an estimated 558 000 MDR/RR-TB patients, of whom only 160 684 (29%) were diagnosed and 139 114 (25%) were initiated on treatment [1]. Before universal drug susceptibility testing (DST), TB patients that were at high risk for MDR-TB (presumptive MDR-TB, erstwhile known as MDR-TB suspects) were prioritized for culture and DST. High pre-diagnosis attrition (presumptive MDR-TB not undergoing culture and DST—17%~90%) and pre-treatment attrition (confirmed MDR-TB patients not initiated on treatment—24%) have been reported [2–16]. Delay from eligibility for DST to diagnosis (diagnosis delay) among patients with presumptive MDR-TB who underwent DST and from diagnosis to treatment initiation (treatment initiation delay) among the confirmed MDR-TB patients initiated on treatment are also major challenges. Among studies published during 2000–15, weighted mean time to treatment from specimen collection was 81 days: it was 108 with phenotypic DST and 38 days with genotypic (rapid molecular tests) DST [8]. Though this has significantly improved over the years, this time interval is still considerable.

Globally, the treatment success rates for MDR-TB are between 55–56% [1,17]. Diagnosis and treatment initiation delay could potentially result in such low treatment success rates. However, a systematic review in 2016 revealed a lack of published evidence globally regarding the association between early treatment initiation after diagnosis and high treatment success rates [18].

China contributes to 13% of the global MDR/RR-TB patients. Seven percent of new TB patients and 24% of previously treated patients have MDR-TB. In 2017, there were an estimated 73 000 MDR/RR-TB patients of whom only 13 069(18%) were diagnosed and 5943(8%) were initiated on treatment [1]. Treatment success rate is poor (40–50%) [1,19]. Median treatment initiation delay after diagnosis in Shanghai (2011–14) was around six months [20]. There is limited information in China regarding diagnosis delay and factors associated with pre-diagnosis and pre-treatment attrition.

With the support of the Global Fund, China initiated a programme for MDR-TB (the Global Fund programmatic management of drug-resistant TB (GFPMDT)) between October 2006 and June 2014 in a phased manner [21]. In this paper, we assessed the attrition and delay in the MDR-TB diagnosis (through phenotypic DST) and treatment pathway and the factors associated.

## Methods

### Study design and population

This was a cohort study involving record review of programme data. All presumptive pulmonary MDR-TB patients belonging to the GFPMDT sites and eligible for DST between October 2006 and June 2013 were included. By June 2013, the GFPMDT covered 67 prefectures across 24 provinces in China (Table 1 and S1 Annex).

**Table 1:** Implementation of GFPMDT project in a phased manner in China from October 2006 to June 2013.

Phase	Period	Newly launched sites*		No. of cumulative sites*	
		Province	Prefecture	Province	Prefecture
Round 5 phase1	Oct 2006-Sep 2008	2	5	2	5
Round 5 phase2	Oct 2008-Sep 2011	4	26	6	31
Round 7	Oct 2008-Sep 2010	6	10	12	41
SSF	Jul 2010-Jun 2013	12	26	24	67

GFPMDT: Global Fund Programmatic Management of drug resistantTuberculosis;SSF: single stream framework

*newly launched and cumulative sites did not included 10 sites launched in 2013 considering the diagnosis and treatment in these sites was not implemented till the end of that year

Genotypic/rapid DST was not introduced into China during GFPMDT project implementation phase.

Presumptive MDR-TB were defined as i) previously treated TB patients which included recurrent TB, return after loss to follow up, treatment after failure and ii) new TB patients that were non-converters at three months of treatment or in close contact with a known MDR-TB patient.

### Setting

#### General setting

China, the world’s most populous country, is a unitary sovereign state in East Asia with a population of over 1.4 billion [22]. It has three levels of sub-national administrative divisions: 34 provinces, 334 prefectures and 2851 counties. The prevalence of all pulmonary and bacteriological confirmed pulmonary TB among population over 15 years of age was 442/100 000 and 116/100 000 respectively [23]. The incidence rate of TB/HIV is estimated to 0.82/100 000. For all the hospitalized patients, HIV test was done routinely, but not free of charge.

#### GFPMDT in China

The launching criteria for PMDT sites included good ‘directly observed treatment–short course’ foundation, sound local government support and willingness to pilot PMDT. Hence, most of the project sites were located in east and middle region where the economic situation was better than the west region.

Patients presented at county-level health institutions (basic management units (BMUs)), where sputum testing was conducted and drug-resistance risk assessment was initiated. Sputum specimen from presumptive MDR-TB patients were transported to prefecture or provincial-level laboratory (culture DST facility) for culture. For specimen with a positive culture result, the proportion method was used to determine the susceptibility of isolates against rifampicin and isoniazid. There was one laboratory register at country / BMU level for routine sputum microscopy. There was a culture DST register at prefecture level laboratory or provincial-level reference laboratory. The culture DST facility shared the DST results with the BMU. All the demographic and clinical data including laboratory test results were recorded in the presumptive MDR-TB register at the BMU.

Patients diagnosed with MDR-TB were referred to the designated hospital at prefecture-level. Prior to initiating treatment, patients underwent a thorough medical examination. They received a standardized treatment regimen for 24 months that consisted of 6–8 months of intensive phase (Pyrazinamide, Amikacin, Levofloxacin, Cycloserine, Prothionamide) and 18 months of continuation phase (Pyrazinamide, Levofloxacin, Cycloserine, Prothionamide) [24].

### Data variables, sources of data and data collection

During January to April 2014, data were collected from the presumptive MDR-TB register at BMU and MDR-TB register at prefecture level designated hospitals. Data on baseline characteristics like province, prefecture, region, age, gender, GFPMDT phase, sputum smear status at TB diagnosis, presumptive MDR-TB criteria, dates of eligibility for DSTand DST result were collected. Among confirmed MDR-TB, date of DST results, treatment initiation (yes/no) and date of treatment initation were extracted. The procedure of data collection and management included the following steps: 1) the local staff from prefectural level filled the questionnaire which was designed at national level; 2) the provincial-level staff conducted data quality assessment and reconciled discrepancies 3) the national-level staff conducted final data review and assessment to eliminate missing data and errors.

### Data management and statistical analysis

Data were single-entered in an MS Excel database in December 2014. The dataset was analyzed using STATA (version 12.1, copyright 1985–2011 StataCorp LP USA).

The following three time intervals were calculated: between eligibility for DST and DST results (diagnosis delay), between diagnosis and treatment initiation (treatment initiation delay), and between eligibility for DST and treatment initiation (total delay). Delays were summarized using median (inter-quartile range-IQR). Pre-diagnosis attrition and pre-treatment attrition were summarized using frequency and proportion.

Predictive modelling using log binomial regression was performed to identify risk factors for attrition. While using the diagnosis delay variable as one of the potential factors associated with pre-treatment attrition, the diagnosis delay was categorized based on quartiles. Linear regression was used to determine the factors associated with delays (one model for each delay: diagnosis delay, treatment initiation delay and total delay). In all the multivariable analyses, variables with unadjusted p<0.2 were included.

The associations in the log binominal models were summarized using relative risks (unadjusted and adjusted–RR and aRR) and 0.95 confidence interval (CI). The associations in linear regression models were summarized using Beta (β) coefficients and 0.95 CI. The β coefficient indicated the adjusted mean difference of delay (in days) between the sub-category of interest and the reference sub-category (negative value meant the adjusted mean value in the category of interest was lower than the reference category; positive value meant the adjusted mean value in the category of interest was higher than the reference category).

As we were dealing with very large numbers of presumptive MDR-TB patients, we assessed the programmatic significance of the β coefficients before assessing the statistical significance. Hence, for the risk factor analysis of diagnosis delay, we considered a β coefficient of at least seven days as programmatically significant association.

### Ethics approval

The study was approved by the Ethics Committee of Chinese Center for Disease Control and Prevention. (number 201807 dated 9 April 2018) and Ethics Advisory Group of the International Union Against Tuberculosis and Lung Disease (The Union), Paris, France (EAG number 23/18 dated 17 April 2018). The study was proposed by National center for TB prevention and control, which was approved by the research department of Chinese Center for Disease Control and Prevention. As this study involved analysis of secondary data, waiver of informed consent was sought and approved by the ethics committees.

## Results

A total of 78 564 presumptive MDR-TB patients were included. Their mean (standard deviation) age was 48.1(18.2) years, 58 392 (74.3%) were males and 43 717 (55.6%) were from east region. Most (74 493, 96.1%) patients were sputum smear positive and 51 635 (65.7%) were ‘new’ TB patients eligible for DST (Table 2).

**Table 2:** Clinical and demographic profile of presumptive MDR-TB patients and confirmed MDR-TB patients under GFPMDT project in China, October 2006-June 2013 [N = 78564].

	Presumptive MDR-TB		Confirmed MDR-TB	
Variable	N	(%)	N	(%)
**Total**	78564	(100)	9283	(100)
Age (years)				
• <15	134	(0.2)	10	(0.1)
• 15–44	33704	(42.9)	4569	(49.2)
• 45–64	28153	(35.8)	3651	(39.3)
• ≥ 65	16573	(21.1)	1053	(11.3)
Gender				
• Male	58392	(74.3)	6864	(73.9)
• Female	20172	(25.7)	2419	(26.1)
Region				
• East	43717	(55.6)	4229	(45.6)
• Middle	30131	(38.4)	4327	(46.6)
• West	4716	(6.0)	727	(7.8)
GFPMDT phase				
• Round 5 phase1	22724	(28.9)	2627	(28.3)
• Round 5 phase2	27401	(34.9)	2663	(28.7)
• Round 7	22263	(28.3)	2766	(29.8)
• SSF	6176	(7.9)	1227	(13.2)
Sputum smear microscopy status				
• Positive	75493	(96.1)	9062	(97.6)
• Negative	3071	(3.9)	221	(2.4)
Presumptive MDR-TB criteria				
• New patients	51635	(65.7)	2331	(25.1)
• Previously treated				
○ Recurrent TB	13024	(16.6)	2878	(31.0)
○ Loss to follow-up	600	(0.8)	129	(1.4)
○ Treatment after failure of new regimen*	3703	(4.7)	1755	(18.9)
○ Others	6557	(8.3)	1453	(15.7)
○ Treatment after failure of ‘previously treated’ regimen^#^	3045	(3.9)	737	(7.9)

GFPMDT: Global Fund Programmatic Management of drug resistanttuberculosis; MDR-TB:Multi drug-resistant tuberculosis, TB:Tuberculosis; SSF: single stream framework

*Category I regimen–TB treatment regimen under national TB programme for newly diagnosed patients;

^#^Category II regimen–TB treatment under national TB programme for previously treated patients.

### Pre-diagnosis and pre-treatment attrition

Of 78 564, 77 372 (98.5%) underwent culture. Of those who tested culture positive, 99.2% (64 852/65 353) underwent DST. Therefore, among 78 564, a total of 2470 (3.1%) underwent pre-diagnosis attrition (1192 specimens did not reach DST laboratory, 777 specimens were contaminated and 501 specimen did not undergo DST despite being culture positive).

**Fig 1:**
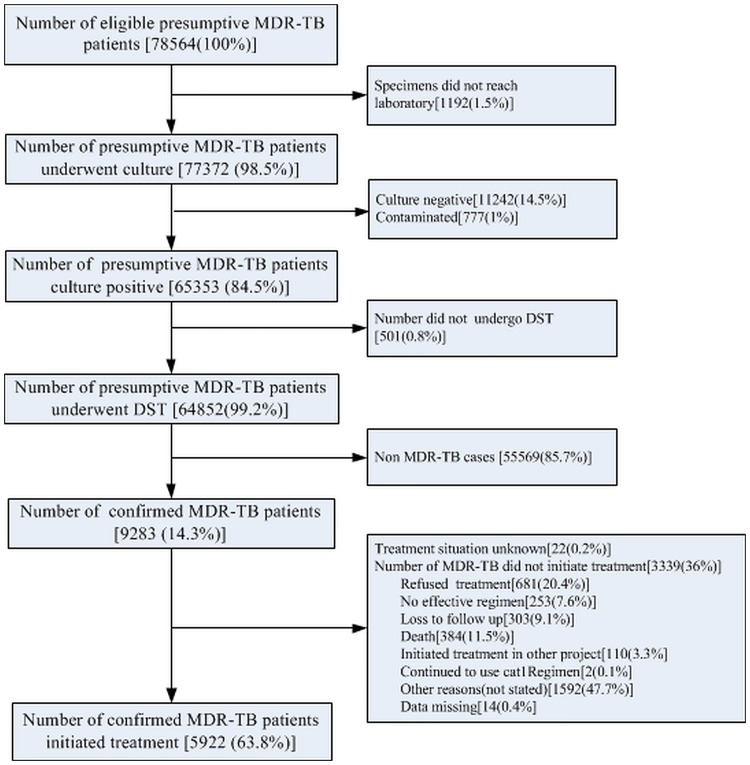
MDR-TB diagnosis and treatment under GFPMDT project in China during October 2006 to December 2013. GFPMDT-Global Fund Programmatic Management of drug resistant tuberculosis;MDR-TB: Multi drug-resistant tuberculosis.

A total of 9283 were diagnosed as MDR-TB and of them 3361 (36.2%) underwent pre-treatment attrition (Fig 1).

### Diagnosis and treatment initiation delays

Median(IQR) diagnosis delay was 84 (64, 114) days; treatment initation delay was 23(6,68) days and total delay was 117(77,187) days (Table 3).

**Table 3:** Time taken (days) for procedures in testing / treatment of presumptive/confirmed MDR-TB patients under GFPMDT project in China, October 2006-June 2013.

Variable	Eligibility–DST [n = 64852]		DST result–treatment initiation [n = 5922]		Eligibility for DST–treatment initiation [n = 5922]	
	Median	(IQR)	Median	(IQR)	Median	(IQR)
**Overall median (IQR)**	84	(64,114)	23	(6,68)	117	(77,187)
Age (years)						
• <15	83	(68,103)	8	(4,53)	84	(75,114)
• 15–44	81	(62,111)	22	(5,62)	112	(73,180)
• 45–64	86	(65,116)	25	(6,75)	120	(81,191)
• ≥ 65	87	(67,117)	26	(9,73)	127	(85,203)
Gender						
• Male	84	(64,114)	23	(6,68)	117	(78,185)
• Female	83	(63,113)	23	(6,66)	116	(76,190)
Region						
• East	83	(63,115)	22	(6,63)	119	(76,183)
• Middle	87	(66,115)	24	(6,78)	119	(79,196)
• West	75	(56,105)	24	(9,56)	100	(75,154)
GFPMDT phase						
• Round 5 phase1	73	(56,98)	35	(10,86)	123	(82,202)
• Round 5 phase2	88	(68,115)	16	(4,48)	111	(73,171)
• Round 7	93	(70,131)	24	(5,76)	119	(69,205)
• SSF	80	(64,103)	24	(9,59)	116	(87,161)
Sputum smear microscopy status						
• Positive	84	(64,114)	23	(6,67)	117	(77,188)
• Negative	78	(56,105)	32	(12,73)	121	(83,167)
Presumptive MDR-TB criteria						
• New patients	85	(65,113)	23	(8,65)	116	(83,182)
• Previously treated						
○ Recurrent TB	75	(62,104)	30	(8,79)	127	(87,203)
○ Loss to follow-up	80	(59,113)	25	(2,67)	101	(78,174)
○ Treatment after failure of category I regimen*	81	(62,111)	16	(2,53)	103	(60,173)
○ Others	84	(62,118)	27	(7,79)	122	(81,189)
○ Treatment after failure of category II regimen^#^	85	(65,113)	17	(3,54)	106	(62,165)

TB: Tuberculosis; MDR-TB: Multidrug-resistant tuberculosis; GFPMDT: Global Fund Programmatic Management of drug resistanttuberculosis; IQR–Interquartile range; SSF: single stream framework

*Category I regimen–TB treatment regimen under national TB programme for newly diagnosed patients;

^#^Category II regimen–TB treatment under national TB programme for previously treated patients.

### Risk factors for pre-treatment attrition

As the pre-diagnosis attrition was very low and not programmatically significant, we are not presenting the risk factor analysis. Pre-treatment attrition was very high. Pre-treatment attrition was significantly i) lower in round 5-phase 2 and round 7 when compared to round 5-phase 1 of GFPMDT phase, ii) higher among elderly when compared to patients in 15–44 year age group, iii) higher in east and west regions when compared to middle region, iv) higher among patients with recurrent TB, treatment after failure of category I/II regimen and previously treated–others, when comared to new TB patients, and v) high among those with long diagnosis delays (dose response relationship seen) (Table 4).

**Table 4:** Association of clinical and socio-demographic factors with pre-treatment attrition among patients diagnosed with MDR-TB under GFPMDT project in China, October 2006-June 2013 [N = 9283].

Variable	Total	Attrition					
	[N]	[n]	(%)^@^	RR	(0.95 CI)	aRR**	(0.95 CI)
**Total**	9283	3361	(36.2)				
Age (years)							
• <15	10	3	(30.0)	0.83	(0.21–3.20)	0.56	(0.14–2.26)
• 15–44	4569	1561	(34.2)	1.00	reference	1.00	reference
• 45–64	3651	1255	(34.4)	1.01	(0.92–1.10)	1.05	(0.96–1.16)
• ≥ 65	**1053**	**542**	**(51.5)**	**2.04**	**(1.79–2.34)** ^	**2.21**	**(1.92–2.55)**^
Gender							
• Male	6864	2521	(36.7)	1.09	(0.99–1.20)	1.05	(0.95–1.16)
• Female	2419	840	(34.7)	1.00	reference	1.00	reference
Region							
• East	**4229**	**1598**	**(37.8)**	**1.16**	**(1.07–1.27)** ^	**1.30**	**(1.18–1.42)**^
• Middle	4327	1484	(34.3)	1.00	reference	1.00	reference
• West	**727**	**279**	**(38.4)**	**1.19**	**(1.02–1.40)** ^	**1.34**	**(1.11–1.62)**^
GFPMDT phase							
• Round 5 phase1	2627	1116	(42.5)	1.00	reference	1.00	reference
• Round 5 phase2	**2663**	**766**	**(28.8)**	**0.55**	**(0.49–0.61)** ^	**0.44**	**(0.39–0.50)**^
• Round 7	**2766**	**948**	**(34.3)**	**0.71**	**(0.63–0.79)** ^	**0.64**	**(0.57–0.72)**^
• SSF	1227	531	(43.3)	1.03	(0.90–1.19)	0.88	(0.76–1.03)
Sputum smear microscopy status							
• Positive	9062	3275	(36.1)	1.00	reference	-	-^^
• Negative	221	86	(38.9)	1.13	(0.86–1.48)	-	-
Presumptive MDR-TB criteria							
• New patients	2331	1124	(48.2)	1.00	reference	1.00	reference
• Previously treated							
○ Recurrent TB	**2878**	**984**	**(34.2)**	**0.56**	**(0.50–0.62)** ^	**0.51**	**(0.46–0.58)**^
○ Loss to follow-up	129	65	(50.4)	1.09	(0.77–1.56)	1.13	(0.78–1.63)
○ Treatment after failure of category I regimen*	**1755**	**411**	**(23.4)**	**0.33**	**(0.29–0.38)** ^	**0.32**	**(0.28–0.37)** ^
○ Others	**1453**	**547**	**(37.7)**	**0.65**	**(0.57–0.74)** ^	**0.59**	**(0.51–0.68)**^
○ Treatment after failure of category II regimen^#^	**737**	**230**	**(31.2)**	**0.49**	**(0.41–0.58)** ^	**0.48**	**(0.40–0.58)**^
Delay between eligibility for DST to DST result in days							
• 1^st^ quartile (<64)	2272	573	(25.2)	1.00	reference	1.00	reference
• 2^nd^quartile (64–83)	**2549**	**1003**	**(39.3)**	**1.92**	**(1.70–2.18)** ^	**1.73**	**(1.52–1.96)**^
• 3^rd^ quartile (84–113)	**2396**	**928**	**(38.7)**	**1.87**	**(1.65–2.13)** ^	**1.82**	**(1.60–2.07)**^
• 4^th^ quartile (≥114)	**2066**	**857**	**(41.5)**	**2.10**	**(1.85–2.39)** ^	**2.06**	**(1.80–2.35)**^

GFPMDT-Global Fund Programmatic Management of drug resistant tuberculosis;MDR-TB: Multi drug-resistant tuberculosis, DST: Drug susceptibility testing; RR: reltative risk; aRR: adjusted relative risk, log binomial regression was performed; SSF: single stream framework

^@^ row percentage

*Category I regimen–TB treatment regimen under national TB programme for newly diagnosed patients;

^#^Category II regimen–TB treatment under national TB programme for previously treated patients

^p<0.05were considered significant difference

**log binomial regression, the confounders that were included in the model were age, sex, phase of implementation, region, presumptive MDR-TB criteria and diagnosis delay categorized based on quartiles

^^sputum status not included in the model as unadjusted p value was >0.20

### Factors associated with delays

The diagnosis delay increased in all the phases when compared to round 5 phase 1. It was significantly i) lower among patients with negative sptum smear microscopy status when compared to those with positive smear, and ii) lower among previously treated patients who were lost to follow-up when compared to new TB patients (Table 5).

**Table 5:** Multivariable linear regression for factors associated with diagnosis delay (between eligibility for DST to DST) among patients with presumptive MDR-TB that underwent DST under GFPMDT project in China, October 2006-June 2013 [N = 68452].

Variables	Beta coefficient**	(95% CI)	p-value
Age (years)			
○ <15	-4.60	(-18.4, 9.21)	0.514
○ 15–44	reference		
○ 45–64	4.79	(3.49, 6.10)	<0.001
○ ≥65	5.62	(4.08, 7.16)	<0.001
Gender			
○ Male	1.57	(0.26, 2.88)	0.019
○ Female	reference		
Region			
○ East	-1.21	(-2.42, -0.01)	0.048
○ Middle	reference		
○ West	**-18.32**	**(-21.17, -15.47)**	**<0.001**^
GFPMDT phase			
○ Round 5 phase1	reference		
○ Round 5 phase2	**14.27**	**(12.8, 15.74)**	**<0.001**^
○ Round 7	**35.73**	**(34.2, 37.26)**	**<0.001**^
○ SSF	**13.24**	**(10.68, 15.79)**	**<0.001**^
Sputum smear microscopy status			
○ Positive	reference		
○ Negative	**-8.91**	**(-12.63, -5.19))**	**<0.001**^
Presumptive MDR-TB criteria			
○ New patients	reference		
○ Previously treated			
○ Recurrent TB	1.28	(-0.29, 2.86)	0.110
○ Loss to follow-up	**-9.38**	**(-15.79, -2.98)**	**<0.001**^
○ Treatment after failure of category I regimen*	-4.13	(-6.86, -1.40)	<0.001
○ Others	5.25	(3.07, 7.43)	<0.001
○ Treatment after failure of category II regimen^#^	-2.34	(-5.78, 1.11)	0.183

GFPMDT-Global Fund Programmatic Management of drug resistanttuberculosis;MDR-TB:Multi drug-resistant tuberculosis, DST:Drug susceptibility testing; SSF: single stream framework; CI–confidence interval

*Category I regimen–TB treatment regimen under national TB programme for newly diagnosed patients;

^#^Category II regimen–TB treatment under national TB programme for previously treated patients

**Linear regression, the confounders that were included in the model were age, sex, phase of implementation, region, sputum positivity, and presumptive MDR-TB criteria;

^Due to the large number of patients, adjusted mean difference of at least seven was considered as programmatically significant after whih statistical significance was assessed (p<0.05)

The treatment initiation delay was significantly i) lower in all phases when compared to round 5 phase 1, ii) lower in east and west region when compared to middle region, iii) higher in elderly when compared to patients in 15–44 year age group, and iv) higher among recurrent TB patients when compared to new TB patients (Table 6).

**Table 6:** Multivariable linear regression for factors associated with treatment initiation delay (between DST and treatment initiation) among bacteriologically-confirmed MDR-TB patients registered for treatment under GFPMDT project in China, October 2006-June 2013 [N = 5922].

Variables	Beta coefficient**	(95% CI)	p-value
Age (years)			
○ <15	-26.71	(-119.88, 66.46)	0.574
○ 15–44	reference		
○ 45–64	5.49	(-1.40, 12.38)	0.120
○ ≥65	**17.26**	**(5.32, 29.19)**	**<0.010**^
Gender			
○ Male	-0.07	(-7.42, 7.28)	0.985
○ Female	reference		
Region			
○ East	**-23.36**	**(-30.16, -16.55)**	**<0.001**^
○ Middle	Reference		
○ West	**-22.36**	**(-36.38, -8.33)**	**<0.001**^
GFPMDT phase			
○ Round 5 phase1	reference		
○ Round 5 phase2	**-35.71**	**(-44.29, -27.14)**	**<0.001**^
○ Round 7	**-14.59**	**(-23.38, -5.79)**	**<0.001**^
○ SSF	**-37.43**	**(-49.65, -25.21)**	**<0.001**^
Sputum smear microscopy status			
○ Positive	reference		
○ Negative	4.92	(-17.04, 26.88)	0.661
Presumptive MDR-TB criteria			
○ New patients	reference		
○ Previously treated			
○ Recurrent TB	**12.90**	**(3.79, 22.01)**	**0.001**^
○ Loss to follow up	7.51	(-24.13, 39.14)	0.642
○ Treatment after failure of category I regimen*	-5.01	(-14.90, 4.87)	0.320
○ Others	5.55	(-5.39, 16.50)	0.320
○ Treatment after failure of category II regimen^#^	-7.34	(-20.49, 5.81)	0.274

GFPMDT-Global Fund Programmatic Management of drug resistanttuberculosis;MDR-TB:Multi drug-resistant tuberculosis, DST:Drug susceptibility testing; SSF: single stream framework; CI–confidence interval

*Category I regimen–TB treatment regimen under national TB programme for newly diagnosed patients;

^#^Category II regimen–TB treatment under national TB programme for previously treated patients

**Linear regression, the confounders that were included in the model were age, sex, phase of implementation, region, sputum positivity, and presumptive MDR-TB criteria;

^p<0.05 were considered significant difference

The total delay was significantly i) lower in round 5 phase 2 and single stream framework when compared to round 5 phase 1, ii) lower in east and west region when compared to middle region, iii) higher in elderly and 45–64 year age group when compared to patients in 15–44 year age group, and iv) higher among recurrent TB patients and treatment after failure in category I/II patients when compared to new TB patients (Table 7).

**Table 7:** Multivariable linear regression for factors associated with total delay (between eligibility for DST and treatment initiation) among bacteriologically-confirmed MDR-TB patients registered for treatment under GFPMDT project in China, October 2006-June 2013 [N = 5922].

Variables	Beta coefficient**	(95% CI)	p-value
Age (years)			
○ <15	-44.70	(-154.42, 65.03)	0.425
○ 15–44	reference		
○ 45–64	**11.27**	**(3.16, 19.39)**	**0.001**^
○ ≥65	**33.30**	**(19.24, 47.35)**	**<0.001**^
Gender			
○ Male	2.90	(-5.76, 11.55)	0.51 2
○ Female	reference		
Region			
○ East	**-16.45**	**(-24.47, -8.43)**	**<0.001**^
○ Middle	reference		
○ West	**-27.93**	**(-44.44, -11.41)**	**<0.001**^
GFPMDT phase			
○ Round 5 phase1	reference		
○ Round 5 phase2	**-38.78**	**(-48.88, -28.68)**	**<0.001**^
○ Round 7	-6.71	(-17.07, 3.65)	0.200
○ SSF	**-33.32**	**(-47.71, -18.93))**	**<0.001**^
Sputum smear microscopy status			
○ Positive	reference		
○ Negative	8.62	(-17.25, 34.48)	0.51 4
Presumptive MDR-TB criteria			
○ New patients	reference		
○ Previously treated			
○ Recurrent TB	**14.90**	**(4.17, 25.63)**	**0.001**^
○ Lossto follow-up	-6.58	(-44.83, 30.68)	0.729
○ Treatment after failure of category I regimen*	**-16.13**	**(-27.77, -4.49))**	**0.001**^
○ Others	4.59	(-8.30, 17.48)	0.485
○ Treatment after failure of category II regimen^#^	**-19.22**	**(-34.70, -3.73)**	**0.015**^

GFPMDT-Global Fund Programmatic Management of drug resistanttuberculosis;MDR-TB:Multi drug-resistant tuberculosis, DST:Drug susceptibility testing; SSF: single stream framework; CI–confidence interval

*Category I regimen–TB treatment regimen under national TB programme for newly diagnosed patients;

^#^Category II regimen–TB treatment under national TB programme for previously treated patients

**Linear regression, the confounders that were included in the model were age, sex, phase of implementation, region, sputum positivity, and presumptive MDR-TB criteria;

^p<0.05 were considered significant difference

## Discussion

This is the first study from China to offer many seminal observations on the MDR-TB diagnosis and treatment pathway—a holistic look at flow of a cohort of presumptive MDR-TB patients which includes pretreatment delay calculation from eligibility for DST and the effect of diagnosis delay on pre-treatment attrition. To the best of our knowledge, ours is the first study to fill the ‘evidence gap’ regarding the association between long diagnosis delay and pre-treatment attrition in the MDR-TB care pathway.

### Limitations

We acknowledge some limitations in this study. First, it is likely that not all the presumptive MDR-TB were identified, therefore the selection bias might have led to an underestimation of attrition, especially pre-diagnosis attrition. Second, the treatment outcomes for a significant cohort of MDR-TB patients was not tracked and collected. Hence, we were not able to study the association between delays and MDR-TB outcomes, evidence for which is limited globally [18]. Third, though this study was unique in calculating the diagnosis delay starting from eligibility for DST, we could not tease out the delay between eligibilty and sputum specimen receipt at DST facility as the date of sputum specimen receipt was not collected. Finally, this being a programme data that is single entered routinely, data entryerrors cannot be ruled out.

### Interpretation of key findings

Limitations notwithstanding, there were some key findings. First, the long total delay was majorly contributed by long diagnosis delay (due to the use of phenotypic DST which happens over two rounds: one round for culture of Mycobacterium tuberculosis (6–8 weeks) and another for DST). The total delay was longer than globally reported average figures with phenotypic DST during 2000–15 (117 vs 108 days), probably due to eligbility of DST (not sputum specimen receipt at DST facility) being the starting point for delay calculation [8]. In our study, while the diagnosis delay increased over years, the treatment initiation delay and total delay decreased over the years (2006–13), which might due to the improvement in timely second line drug supply.

Second, the pre-treatment attrition was very high when compared to the global figures of 24% during 2000–15 (reduced to 13% in 2017) [1,8]. Almost half of the patients who underwent pre-treatment attrition either refused treatment or were lost to follow-up or died (Fig 1). Patients refused treatment possibly because of a long two month period of hospitalization during treatment at prefecture level. Shortage of second line drugs due to the long turnaround time of international procurement was a possible reason for pre-treatment attrition during the initial phases.

Third, long diagnosis delay contributed to pre-treatment attrition possibly due to patients being very sick at diagnosis to move to a prefecture level facility.

Finally, within the project, around two-thirds of the presumptive MDR-TB patients were new TB patients. In India, majority of the patients were previously treated patients and non-identification of presumptive MDR-TB contributed largely to pre-diagnosis attrition [4–7,15,16]. In our study, though the pre-diagnosis attrition among identified presumptive patients was low, non-identification of large numbers of previously treated patients as presumptive MDR-TB cannot be ruled out. This finding also corroborates with overall low MDR-TB detection rates (8%) as a proportion of estimated MDR-TB in China in 2013 [19].

### Implications for policy and practice

There are important implications for China. First, though diagnosis delay was increasing over time period, we expect that by the introduction of rapid molecular DST, we should be able to reduce this delay which will in turn reduce pre-treatment attrition, the total delay and potentially result in better outcomes. China has recently updated the national TB guidelines (end of 2018). Along with the world health organization guidelines [25], this study provides evidence base to expand the use of rapid molecular tests. China now plans to expand the availability of rapid molecular tests at BMU level in at least 80% counties by 2020. In addition to the high risk group described in this study, the national programme plans to expand its use to all sputum positive pulmonary TB patients.

Second, to reduce the pre-treatment attrition and treatment initiation delays, we recommend decentralized MDR-TB treatment. This may be tried at least for those patients who are not ill at diagnosis [16]. A community-based MDR-TB care model may be tried to improve treatment initiation as reported in Myanmar [26]. There is a need to move towards reducing the mandatory inpatient care for two months which could be a potential barrier to take treatment. Shorter treatment regimen may be further tried in a select group of patients The shorter regimens have shown high treatment success rates in operational settings [27].

Third, low contribution of previously treated patients in the presumptive MDR-TB cohort indicates that there is a critical need to assess this situation nationally in China. The national estimates of presumptive MDR-TB patients based on the presumptive criteria and the number of TB patients that actually undergo culture and phenotypic DST and/or rapid molecular testing (using national TB surveillance data) may be compared.

Finally, a qualitative systematic enquiry is recommended to understand why certain risk groups are more prone to experience attrition and longer delays [28].

## Conclusion

In this MDR-TB care pathway from the Global Fund PMDT project in China (2006–13), we holistically documented the attrition, delays and their associated factors. Not only does this study emphasize the importance of early treatment initiation but also brings the focus on early diagnosis by testing patients as soon as they are eligible which can reduce pre-treatment attrition as well as potentially improve treatment outcomes. As China prepares to expand the coverage of rapid molecular DST at the level of counties, similar studies are recommended in future to monitor the reduction of delay and attrition along the MDR-TB care pathway, and the effect of pre-treatment delays (starting from DST eligibility) on treatment outcomes. This is vital if we are to end TB in China and globally by 2035 [29].

## Supporting information

S1 Annex Timeline and strategies under China Global Fund programmatic management of drug-resistant tuberculosis programme scale-up (2006–13). (TIF)

S2 Annex Dataset containing data and codebook. (XLSX)
