# Supplementary figures and images for: Attrition and delays before treatment initiation among patients with MDR-TB in China (2006-13): Magnitude and risk factors
Source: PLoS One. 2019 Apr 8;14(4):e0214943. doi: 10.1371/journal.pone.0214943 (PMC6453446; doi:10.1371/journal.pone.0214943)

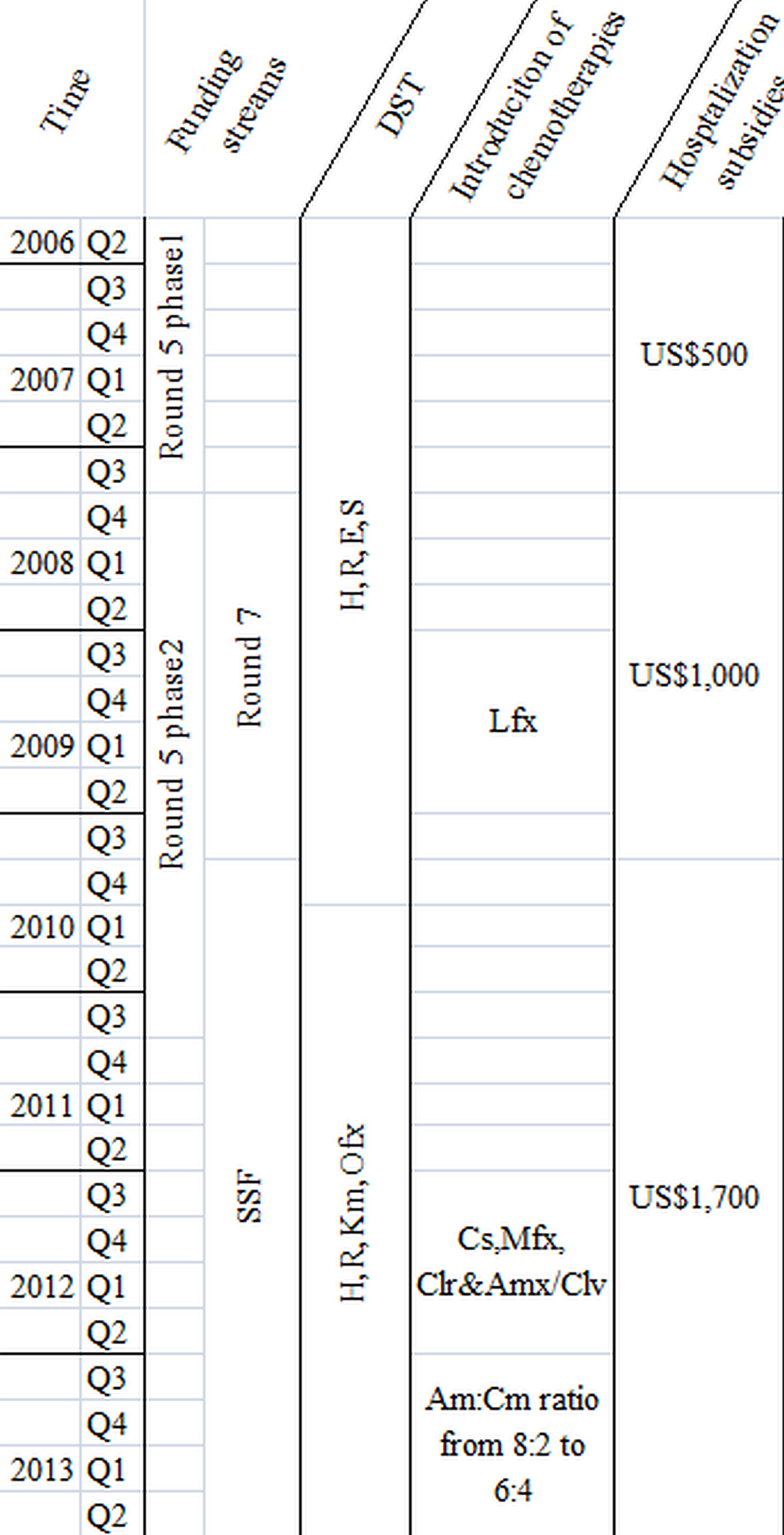

Supplement: S1 Annex — (TIF) [file pone.0214943.s001.tif]
